# Rethinking ADHD intervention trials: feasibility testing of two treatments and a methodology

**DOI:** 10.1007/s00431-019-03374-z

**Published:** 2019-04-24

**Authors:** Philippa Fibert, Tessa Peasgood, Clare Relton

**Affiliations:** 1 School of Health and Related Research, University of Sheffield, Regent Coutrt, 30 Regent Street, Sheffield, S1 4DA UK; 2 Health Economics and Decision Science, West Court, Mappin Street, Sheffield, S1 4DP UK; 3 Pragmatic Clinical Trials Unit, Centre for Primary Care and Public Health, Queen Mary’s University, 58 Turner Street, London, UK

**Keywords:** Attention deficit hyperactivity disorder, Homoeopathy, Nutritional therapy, Trials within cohorts

## Abstract

Attention deficit hyperactivity disorder (ADHD) is a lifelong condition associated with considerable costs. The long-term effectiveness and acceptability of treatments to improve outcomes remains in doubt. Long-term trials are needed comparing interventions with standard care and each other. The Sheffield Treatments for ADHD Research (STAR) project used the Trials within Cohorts (TwiCs) approach. A cohort of children with ADHD was recruited and outcomes collected from carers and teachers. A random selection was offered treatment by homoeopaths (hom) or nutritional therapists (NT). Their outcomes (Conners Global ADHD Index) were compared with those not offered interventions. The feasibility of the methods and interventions was assessed. The TwiCs approach was feasible with modifications. 144 participants were recruited to the cohort, 83 offered treatment, 72 accepted, and 50 attended 1+ appointments. Results according to carers assessments at 6 months were as follows: *t* = 1.08, *p* = .28 (− 1.48, 4.81) SMD .425 (hom); *t* = 1.71, *p* = .09 (− .347, 5.89), SMD = .388 (NT). Teachers’ responses were too few and unstable. No serious treatment adverse events occurred.

*Conclusion*: the STAR project demonstrated the feasibility of the TwiCs approach for testing interventions for children with ADHD.

**Table:** 

**What is Known:** • *Attention deficit hyperactivity disorder (ADHD) is a lifelong condition associated with considerable costs to ADHD stakeholders. Children are at risk of negative outcomes and in need of pre-emptive strategies* • *The long-term effectiveness and acceptability of recommended treatments to improve outcomes remains in doubt*	
**What is New:** • *A small-scale test of the design demonstrated that the Trials within Cohorts (TwiCs) approach is feasible and can make a useful contribution regarding testing the effectiveness of interventions for children with ADHD to improve long-term negative outcomes* • *Treatment by homoeopaths and nutritional therapists may offer novel opportunities to improve outcomes.*

**What is Known:**

• *Attention deficit hyperactivity disorder (ADHD) is a lifelong condition associated with considerable costs to ADHD stakeholders. Children are at risk of negative outcomes and in need of pre-emptive strategies*

• *The long-term effectiveness and acceptability of recommended treatments to improve outcomes remains in doubt*

**What is New:**

• *A small-scale test of the design demonstrated that the Trials within Cohorts (TwiCs) approach is feasible and can make a useful contribution regarding testing the effectiveness of interventions for children with ADHD to improve long-term negative outcomes*

• *Treatment by homoeopaths and nutritional therapists may offer novel opportunities to improve outcomes.*

## Introduction

Attention deficit hyperactivity disorder (ADHD) is a lifelong condition associated with considerable costs to a significant proportion of those with ADHD, their carers, and society. It is a leading cause of child referrals to mental health services, and a major risk factor for early criminality, poor educational outcomes, school drop-out, and expulsion.

Although short-term effects (12 weeks) are reported for pharmaceutical medications, and behavioural interventions during participation, the long-term effectiveness and acceptability of mainstream recommended treatments to improve outcomes remains in doubt [5, 29–31]. Medications are not well tolerated and associated with side effects such as reduced growth over the long term [30]; nausea, reduced appetite, sleep problems in the short term [21]; and long-term effects are not established in the few trials conducted. The effects found during participation in behavioural interventions are contested due to lack of blinded outcomes, and lack of improvement in core ADHD symptoms [35]. Other interventions are tried by carers [3, 10–12], but their effectiveness has not yet been rigorously assessed.

If outcomes for those with ADHD, their carers, and society are to improve, there is a need to rigorously evaluate the long-term effectiveness and acceptability of interventions by conducting long-term trials comparing promising interventions with standard care and with each other. The standard approach is to conduct short, stand-alone trials comparing an intervention with a placebo or another intervention. Trialling interventions of different types, one at a time by different research teams, using different designs, comparators, inclusion criteria and measurements, is financially and scientifically inefficient. It makes interventions difficult to compare, and may not inform whether they improve long-term outcomes.

In order to rigorously and comparatively assess the effectiveness and acceptability of interventions which might improve outcomes for those with ADHD, their carers, and society, we piloted a novel, alternative approach to randomised controlled trial (RCT) design—the Trials within Cohorts (TwiCs) approach. The TwiCs design was developed to address shortcomings associated with conducting RCTs, such as recruitment, ethics, patient preferences, and treatment comparisons, and more closely replicate real-world routine health care [23]. It is being applied globally in at-risk cohorts of children particularly where the aim is to improve long-term outcomes (https://www.twics.global/use-of-the-design).

The TwiCs approach entails recruitment of a large observational cohort and regular measurement of their outcomes. For each RCT, eligible participants are identified from the cohort and some randomly selected to be offered the trial intervention(s). Their outcomes are compared with those of eligible participants not selected (that is, receiving usual care). The approach enables reliable comparisons because all treatments have the same parameters and risk of bias, are conducted within the same population, and measure the same outcomes. Currently, the two main treatment categories for ADHD (behavioural and pharmaceutical) are difficult to compare because they differ in these aspects.

The Sheffield Treatments for ADHD Research (STAR) Project was set up with the aim of improving outcomes for those with ADHD (https://www.facebook.com/starsheffieldADHD, www.starsheffield.com). This article reports the results of Stage 1 of the STAR project, which was to assess the feasibility of the TwiCs trial design to provide suitable information for stakeholders to enable evaluation of the clinical and cost effectiveness of some treatments for ADHD identified as being used by carers. This was done by conducting a small-scale test of the methods and procedures: a three-armed internal pilot trial of the clinical and cost effectiveness of (a) the offer of adjunctive treatment by homoeopaths and (b) the offer of adjunctive treatment by nutritional therapists, compared with (c) treatment as usual [7] (ISRCTN17723526).

The objectives were to assess the feasibility of recruiting a cohort of children with a diagnosis of ADHD to time and target; test the feasibility and acceptability of the study design; the feasibility, deliverability, safety, acceptability, preliminary clinical, and cost effectiveness of the interventions; the suitability, acceptability, and deliverability of the outcome measures; and inform the sample size calculation for the full trial. Key feasibility criteria and parameters are summarised in Table 1. Reporting follows Consort guidelines, using the extension for the reporting of pragmatic trials [34].

**Table 1:** Feasibility criterion and results

Criteria (section)	Measurement: criteria parameters	Results	Continuation to a full trial yes/no/recommendations
Recruitment to cohort rates	# recruited in 2 years: % recruited /sample size estimation	144 recruited in 1 year	Yes
Recruitment to treatment rates	% of eligible participants recruiting to the cohort accepting an offer: At least 30%	23/41 (56%) hom; 27/42 (64%) NT	Yes
Treatment effects (SMD baseline-6 months)	SMD CGI: mean = < .3 in those implementing a therapy	36 hom; .55 NT	Yes
Treatment effects (clinical significance)	CGI *T* score: 5 percentiles		Use of *T* scores not feasible due to ceiling effects. SMD (above) used instead
Attrition. Cohort	# CQ’s returned at 6 months: at least 30%	70% (88/124) 6-month questionnaires returned	Yes
Attrition. Consultations	# consultations attended: 70% of participants accepting intervention attend at least 3 consultations	39/42 NT; 33/41 homaccepted the offer.	No
		18/39 (46%) NT; 17/33 (52%) hom had 3+ consults.	
Acceptability of TQ	#TQs completed at baseline and 6 months: # of reminders needed; # email/telephone/paper responses. Adjustment of measure, collection method, and trial parameters	54 (43.5%) completed at baseline. 46 (37.1%) completed at 6 months.	Current methods not feasible: more reminders by a variety of methods needed
Acceptability of CQ	# reminders needed: adjustment of measure, collection method, and trial parameters	Maximum reminders: 3 emails, 1 text, 1 letter.	£10 Boots vouchers introduced improved return rate
Adverse events	Clinician records: no intervention-related severe adverse events, as defined by CTCAE (2010) and EC (2011) guidelines.	No severe events	Yes
Appropriate outcome measurement–CQ	# missing items: adjustment of measure.	5 items missing from paper questionnaires	Continue using on-line questionnaires
Recruitment of therapists	# recruited fulfilling criteria: at least 2 for each therapy	8 therapists (hom); 4 therapists (NT)	One (hom) dropped out/unsuitable. Two (hom) using a receptionist and one (NT) only using email made poor contact with participants.
Suitability of consultation venues/mode	ANCOVA (venue/mode as variable): No venue/mode to have statistically significant impact on treatment effect	This could not be calculated as some therapists used several modes.	
Statistical analysis	ANCOVA: meets assumptions	Outliers not improved with transformation	Regression analysis used. Assumptions met.

## Methods

### Recruitment of the STAR cohort

**Fig. 1:**
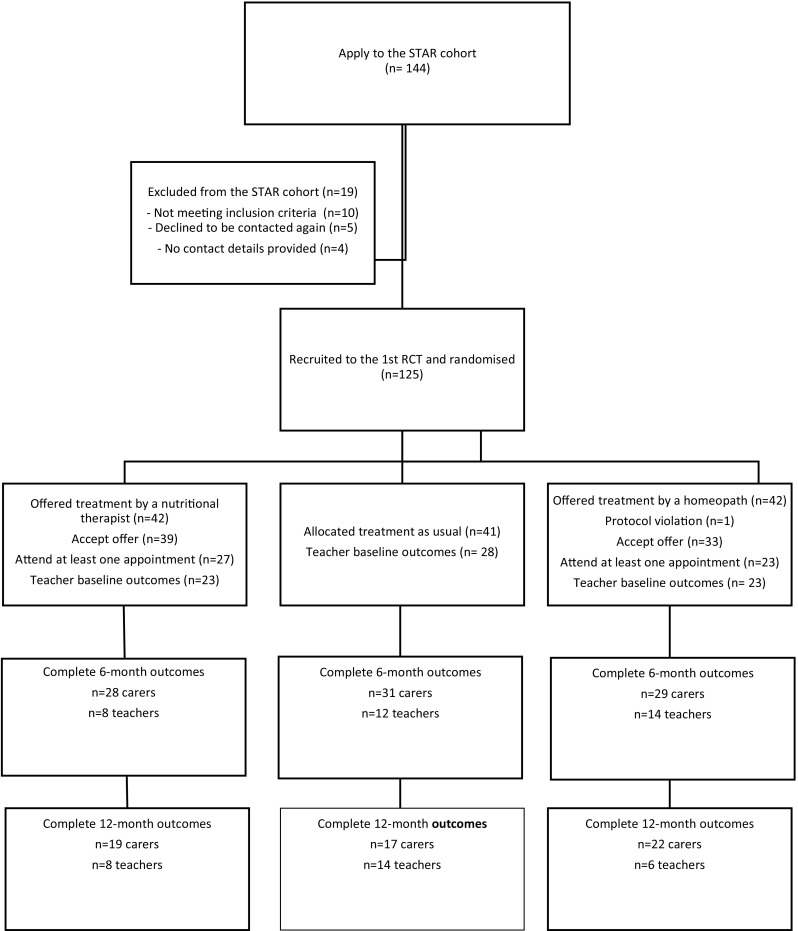
Study progression

Figure 1 describes the study progression. Children with ADHD were recruited to an observational cohort from a broad variety of sources. Cohort inclusion criteria were children aged 5–18 (inclusive) with a carer-reported diagnosis of ADHD and Conners’ Global ADHD Index (CGI) *T* score of at least 55 [4], and any co-morbidities. Exclusion criteria were children with terminal or life-threatening conditions, and families where English was not written or spoken.

### Collection of outcomes

Outcomes were collected from carers and children’s schools at 0, 6, and 12 months via questionnaires. Carers could opt to provide details of their child’s school and if they did, school questionnaires were sent to the head teachers asking someone who knew the child well to complete the questionnaire. Carers were reminded to return the questionnaires via 3 monthly emails and test messages. Teacher outcomes were requested just once by post.

The primary outcome measure was the ten item CGI with sub-scores restlessness/impulsivity (7 items) and emotional lability (3 items) [23] at 6 months. Carers also completed a child health–related quality of life measure (CHU 9D) [26].

### The pilot RCT

For the first trials embedded in the STAR cohort, a proportion of eligible participants were randomly selected and offered treatment by homoeopaths or by nutritional therapists. Inclusion criteria for the pilot trial were a carer-reported ADHD diagnosis and CGI *T* score of 65+. Exclusion criteria were children currently receiving treatment by a homoeopath or a nutritional therapist.

Randomisation was performed by an independent statistician at the University of Sheffield in blocks of 6 with stratifying factors age, medication status, and ADHD severity. The randomisation list was housed in the locked drawer of another independent statistician who randomly assigned participants to one of the three groups.

Those randomised to usual care were not informed that they had not been selected for a treatment. Those selected to be offered treatment were sent a letter offering them 1 year of that treatment, a brief description of what to expect, and asking carers to confirm their child was happy to participate. If both consented, their designated therapist arranged appointments with them. If therapists failed three times to make contact with participants, they were deemed non-responders.

Eight therapists (4 nutritional therapists and 4 homoeopaths) were initially recruited via Wellforce Integrated Medicine Centre in Sheffield, UK. Consultations mirrored usual practice: they took place in therapist’s usual treatment venues; were delivered according to usual modes (face to face, telephone, or on-line); missed appointments were rebooked; and times between consultations and number of consultations varied. Before the trial, therapists attended workshops in management of ADHD, and identification and management of abuse and safeguarding of children.

Participants’ doctors were sent letters explaining that their patients were participating in a trial, describing the ethical approvals and safeguards in place, confirming that interventions should not interfere with pharmaceutical medication, and that participants were advised to continue with their current treatments. Adverse events were recorded according to the Common Terminology Criteria for Adverse Events [32] guidelines and European Commission guidelines [6] and independently assessed by two researchers.

### Statistical analysis

IBM SPSS 21 statistical software was used. Tests were two-tailed with significance level (alpha) set to 5%, and 95% confidence intervals were presented. Each treatment was compared with usual care. Change scores were calculated by subtracting 6-month scores from baseline scores (lower scores indicate better outcomes).

Preference weights were added to health-related quality of life measure CHU 9D, derived from the application of the standard gamble method from 300 members of the UK adult population [27]. This estimates the importance of change in one item versus change in another and versus extending years of life, and gives a score where 0 is equivalent to being dead and 1 represents full health.

The primary outcome used intention to treat (ITT) analysis, whereby all participants offered treatment remained within the treatment group regardless of whether or not they took up the offer of treatment. Secondary analyses explored the effect of having a treatment on the outcome to inform feasibility criterion.

Statistical testing was exploratory since the pilot study was not powered to detect statistical differences. Regression analysis explored the predictive power of the offer of treatment, with analyses controlling for the effects of gender, ADHD severity, and age. Standardised mean differences (SMDs) (Cohen’s *d*) explored the magnitude of the clinical effect and provided estimates for the sample size required in the full trial.

## Results

### Recruitment and participation in the STAR cohort

A total of 144 participants completed the carer questionnaire between September 2015 and 2016. 19/144 (13%) did not meet the cohort inclusion criteria or could not be included (no contact details or declining to be contacted again).

**Fig. 2:**
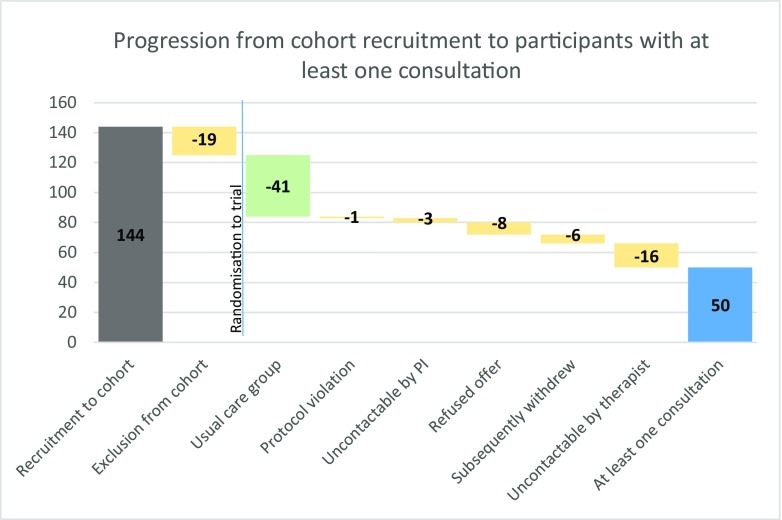
Participant progression from cohort recruitment to first consultation

A total of 125 cohort participants were eligible for the trial and randomised: 42 to treatment by homoeopaths (hom), 42 to treatment by nutritional therapists (NT), and 41 to remain in the usual care group (TAU). One child randomised to treatment by a homoeopath was found to be very sick and awaiting a liver transplant when telephoned to be allocated a therapist. Since this was in violation of the protocol, he was withdrawn from the trial. Seventy-two of the 83 participants offered a treatment accepted it. 50/72 (*n* = 23 hom; *n* = 27 NT) took up their offer and had at least one consultation. Figure 2 describes reasons for non-participation.

### Questionnaire return

A total of 124 baseline Carer Questionnaires, 88 6-month questionnaires, and 58 12-month questionnaires were returned. Of those randomised to a treatment, the majority of returned 6-month (20/29 hom; 24/28 NT) and 12-month (16/22 hom; 16/19 NT) questionnaires were from those who had that treatment. There were just five instances of missing data in the few paper Carer Questionnaires. Last observation carried forward was used to impute the missing data in these few instances.

Teacher outcomes were potentially available from a maximum of 100 teachers, since 20 carers refused permission for their child’s school to be contacted and 4 children were home schooled. Seventy-two baseline, 34 6-month, and 58 12-month Teacher Questionnaires were returned. Schools did not return questionnaires consistently: 31 paired baseline and 6-month questionnaires, 14 paired 6 and 12-month questionnaires, and 21 paired baseline and 12-month questionnaires were returned. Thirty-five percent of paired questionnaires were returned by different teachers.

### The pilot RCT

Randomised groups were similar regarding age and medication status. Fewer with an autism diagnosis were offered NT. Those participants who received a treatment were less likely (but non-significantly so according to log-linear analysis) to have autism or be on ADHD medication, and to have more severe ADHD (Table 2).

**Table 2:** Demographic and clinical characteristics of study participants according to those offered interventions, those receiving interventions; and those continuing with usual care, at baseline, 6, and 12 months

	Hom offered	Hom received	NT offered	NT received	TAU	All
Sample size (i.e. questionnaire return)						
Baseline	*N* = 41	*N* = 23	*N* = 42	*N* = 27	*N* = 41	*N* = 124
6 months	*N* = 29	*N* = 20	*N* = 28	*N* = 23	*N* = 31	*N* = 88
12 months	*N* = 22	*N* = 16	*N* = 19	*N* = 16	*N* = 17	*N* = 58
Mean (standard deviation)						
Age	10.17 (2.42)	9.78 (2.22)	10.05 (2.46)	10.22 (2.68)	10.41 (2.49)	10.21 (2.44)
Female	8 (19.5%)	5 (21.7%)	7 (16.6%)	4 (14.8%)	5 (12.2%)	20 (16.1%)
Taking pharmaceutical medication	28 (68%)	13 (56.5%)	29 (69%)	20 (74%)	27 (65.9%)	84 (67.7%)
Have autism	13 (10%)	6 (5%)	9 (7%)	6 (5%)	15 (12%)	37 (30%
CGI baseline	23.9 (3.56)	23.48 (3.27)	23.9 (3.56)	23.4 (3.87)	22.27 (4.62)	23.1 (4.17)
CGI 6 months	20.0 (6.15)	19.65 (5.83)	18.82 (5.59)	18.48 (5.27)	20.06 (5.29)	19.65 (5.64)
CGI 12 months	19.91 (6.05)	19.63 (5.8)	19.84 (5.5)	19.63 (5.6)	17.88 (6.7)	19.27 (6.03)
Restless/impulsive baseline	17.61 (2.63)	17.3 (2.55)	17.4 (3.09)	17.67 (3)	16.98 (3.41)	17.33 (3.05)
Restless/impulsive 6 months	15.28 (4.6)	14.9 (4.35)	13.68 (3.89)	13.39 (3.6)	15.19 (3.73)	14.74 (4.1)
Restless/impulsive 12 months	15.18 (4.14)	14.88 (3.56)	14.42 (4.14)	14.25 (4.37)	13.71 (5.24)	14.5 (4.44)
Emotional lability baseline	6.39 (1.56)	6.17 (1.59)	5.67 (2.09)	5.74 (1.99)	5.29 (1.93)	5.29 (1.92)
Emotional lability 6 months	4.72 (2.15)	4.75 (2.14)	5.14 (2.1)	5.09 (2.09)	4.87 (2.28)	4.91 (2.16)
Emotional lability 12 months	4.73 (2.43)	4.75 (2.59)	5.42 (2.19)	5.38 (2.13)	4.18 (2.67)	4.79 (2.44)
CHU9D utility scores baseline	.679 (.114)	.676 (.122)	.696 (.106)	.695 (.121)	.708 (.104)	.694 (.108)
CHU9D utility scores 6 months	.708 (.137)	.684 (.149)	.759 (.121)	.769 (.116)	.708 (.130)	.724 (.130)
CHU9D utility scores 12 months	.875 (.151)	.837 (.165)	.903 (.138)	.875 (.148)	.885 (.141)	.888 (.143)

### Data analysis of carer ratings

Primary outcome (ITT analysis of CGI total score at 6 months) results are presented in Table 3 together with per protocol results. Data met assumptions for conducting parametric analyses. When CGI total change score was the dependent variable and group (hom or NT), age, gender, and ADHD severity the covariates, the model explained a significant amount of the variance in CGI change score due to the highly significant influence of ADHD severity (*t* = 4.225, *p* < .001). Neither treatment group, age, nor gender explained a significant amount of variance. Standardised mean differences (SMDs) were as follows: hom .425; and NT .388.

**Table 3:** Primary outcome results: ITT and per protocol regression analyses baseline—6 months, and effect sizes, of Conners Global Index and Health-Related Quality of Life measure CHU-9D, according to carers and teachers

Outcome	Completer	ITT/per protocol	*R* ^2^	Hom (*n* = 29 (ITT), *n* = 20 (received)				NT (*n* = 28 (ITT), *n* = 24 received)			
				*B*^a^ (S.E.)	*t*	C.I.	Effect size^b^	*B* (S.E.)	*t*	C.I.	Effect size^b^
CGI	Carer	ITT	.215	1.7 (1.57)	1.08, *p* = .28	− 1.48, 4.81	.425^**#**^	2.66 (1.55)	1.71, *p* = .09	− .347, 5.89	.388
		Received	.207	1.65 (1.72)	.96, *p* = .34	− 1.84, 5.06	.356	3.05 (1.6)	1.9, *p* = .062	− .044, 6.44	.55^**#**^
	Teacher	ITT	.290	.58 (2.89)	.2, *p* = .84	− 5.37, 6.53	.069	4.11 (3.14)	1.31, *p* = .2	− 2.35, 10.58	.39
		Received	.176	.572 (3.16)	.18, *p* = .86	− 6.09, 7.24	.109	− 1.98 (4.15)	− .477, *p* = .64	− 10.74, 6.78	−.504
Restless-impulsive	Carer	ITT	.171	.437 (1.19)	.368, *p* = .71	− 1.9, 2.8	.198	2.13 (1.18)	1.8, *p* = .075	− .22, 4.47	.418^**#**^
		Received	.181	.594 (1.3)	.456, *p* = .65	− 2.0, 3.19	.172	2.59 (1.22)	2.11, *p* = .038*	.15, 5.03	.623^**#**^
	Teacher	ITT	.264	.176 (2.39)	.074, *p* = .94	− 4.75, 5.1	.016	3.5 (2.6)	1.35, *p* = .19	− 1.86, 8.85	.421^**#**^
		Per protocol	.144	.456 (2.74)	.166, *p* = .87	− 5.33, 6.24	.097	− .824 (3.6)	− .229, *p* = .82	− 8.42, 6.78	− .339
Emotional lability	Carer	ITT	.230	1.23 (.59)	2.09, *p* = .04*	.06, 2.4	.793^**#**^	.648 (.58)	1.11, *p* = .27	− .51, 1.81	.269
		Per protocol	.213	1.02 (.64)	1.59, *p* = .12	− .27, 2.3	.679^**#**^	.612 (.6)	1.01, *p* = .31	− .59, 1.82	.325
	Teacher	ITT	.251	.403 (.75)	.537, *p* = .6	− 1.14, 1.95	.25	.615 (.82)	.754, *p* = .46	− 1.07, 2.3	.195
		Per protocol	.209	.117 (.737)	.158, *p* = .88	− 1.44, 1.67	.117	− 1.16 (.968)	− 1.19, *p* = .25	− 3.2, .89	− .93^**#**^
CHU 9D	Carer	ITT	.102	.057 (.031)	1.84, *p* = .069	− .12, .01	.43^**#**^	.084 (.031)	2.73, *p* = .008*	− .15, − .023	1.1^**#**^
		Per protocol	.122	.05 (.035)	1.42, *p* = .16	− .12, .02	.3	.094 (.03)	2.84, *p* = .006*	− .16, − .03	1.19^**#**^

Total number of observations = 88. Analysis controls for age, gender, and ADHD severity

*Outcome reached two-tailed statistical significance level < .05

^#^Effect size < .4

^a^Unstandardised coefficient

^b^Effect size based on Cohen’s *d*

The variance in both CGI subscale change scores was explained by the influence of ADHD severity, and treatment by a homoeopath also explained a significant amount of the variance in the emotional dysregulation change score (*t* = 2.09, *p* = .04). SMD = .793 and treatment by nutritional therapists neared statistical significance for the restlessness/impulsivity change score (*t* = 1.8, *p* = .075). SMD = .418.

Although improvements sustained at 6 months in treatment groups remained stable at 12 months, some participants in the usual care group registered large improvements at 12 months (Table 2).

### Teacher ratings

Testing may not be valid given the very small number of paired questionnaire returns, compounded by additional uncertainty since 1/3 were completed by different teachers. At 6 months, the positive direction of improvements in NT according to ITT analysis (SMD = .39) became a negative direction when only those accessing treatment were considered (SMD = − .504). None of the covariates entered into the model explained a significant amount of the variance in teacher-rated CGI total or sub-scores (Table 3).

### Health-related quality of life

At 6 months, treatment groups’ health-related quality of life (HRQOL) improved whilst that of those continuing with their usual care did not. Treatment by a nutritional therapist reached statistical significance: *t* = 2.73, *p* = .008, SMD = 1.1, whilst treatment by a homoeopath did not: *t* = 1.84, *p* = .069, SMD hom = .43 (Table 3). At 12 months, HRQOL continued to improve for those in the treatment groups, and there was also a sharp increase in the HRQOL of those in the control group.

### Costs

A total of 240 sessions were attended by participants (124 hom; 124 NT), of which the majority (91 hom; 81 NT) were during the first 6 months. The mean cost of consultations (including the cost of homoeopathic medicines, nutritional supplements, room hire, and postage) for 1 year was £169.31 (hom) and £553.19 (NT). The increased cost of nutritional therapy compared with homoeopathy was due to the cost of nutritional supplements (total cost £16,043 over 1 year).

### Feasibility

Table 1 summarises feasibility criteria results. The design was acceptable, with the trial receiving ethical approval and considered low risk by University of Sheffield health care research governance procedures. The decision to not seek UK National Health Service (NHS) ethical approval was challenged, but the trial sponsor and ethics committee confirmed that it was not required if NHS premises were not used.

A broadly representative ADHD cohort was recruited: 62% had co-diagnoses; 62% were taking ADHD medication; 67% had made at least one visit to the doctor; 60% at least one visit to hospital; one third were taking sleep medications; 95% of families had accessed or were accessing a parenting class; 46% of families had visited or were visiting psychologists; 56% of children had a teaching assistant, of whom 22% had one full time; 7% of families were involved with social workers; 6% of children had been excluded; and 5% involved with the police.

Recruitment to the cohort was feasible, with sufficient numbers speedily recruited. The most successful means was ADHD support groups. There was minimal uptake from schools approached. It was not possible to recruit from nationally funded ADHD facilities because NHS ethical approval was required.

Carer Questionnaire 6-month return rates were initially very low, despite reminders, but improved after addition of a £10 voucher incentive although continued to be low in those who had not accepted the offer of a treatment. Carer-rated treatment effects were sufficient according to the a priori specified criteria. Sufficient paired outcomes were available to inform the sample size calculation for the full trial. Assuming an effect size of .4 for the primary outcome (CGI total score) and 80% power for equal sample sizes, the responses of 100 participants per arm will be required. Forty percent attrition needs to be allowed for, therefore requiring recruitment of 166 participants per arm.

A single request, with no reminders, was made for Teacher Questionnaires. This was unfeasible, since they were poorly and inconsistently returned. There was little missing data indicating that outcome completion methods were manageable.

Treatment acceptance rates were feasible, but non-take up of the offer—post-acceptance—was greater than anticipated, meaning crossover from treatment to usual care was high, and attrition from consultations therefore also high. Uptake was affected by therapist’s contacting strategies: most therapists used a variety of modes and had contacting rates of 60% +; however, two therapists using a clinic receptionist had contacting rates of 33% and 25%, and one therapist relying only on email a contact rate of 33%.

Consultations were conducted at complementary health clinics, participant’s homes, by telephone, or on-line. They consisted of an initial consultation of 1½ h and up to seven follow-up appointments of 30–40 min at 4–6-week intervals.

The initial homoeopathic consultation focused on building up a complete picture of the participant, asking about medical history, life events, likes and dislikes, lifestyle, behaviour, and personality. Prescription of homoeopathic medicines was made by matching the composite of symptoms and patient characteristics with those of an appropriate medicine. At follow-up consultations, carer and child were asked about changes in symptoms and prescriptions continued with or changed according to the response.

The initial nutritional therapy consultation asked about diet preferences, types of food eaten, typical daily diet, food intolerances, lifestyle, stressors, family history, diagnoses, health concerns, and diet-related symptoms. Therapists then provided participants with a summary sheet with a range of individually tailored options and meal suggestions. At subsequent consultations, the plan was reviewed and revised dependent on the family’s ability to assimilate and put suggestions into practice. Options included the following: elimination diets (e.g. gluten free and/or casein free); reducing intake of known problematic substances (e.g. food colourings, sugars); increasing intake of healthy foods (e.g. oily fish, nuts, seeds, fruit, vegetables); substituting less healthy foods with healthier ones; balancing blood sugar; improving drinks/fluids intake; lifestyle advice (e.g. sleep, activity, purpose, relaxation, time outdoors); specific dietary interventions for symptoms; and supplementation (e.g. polyunsaturated fatty acids, multi-vitamins, pro-biotics).

Therapists conducting on-line consultations commented that they liked them, and observed that they may have improved attendance. Nutritional therapists offered carers the choice about whether to attend with their child and most chose not to. Homoeopaths preferred to see both carer and child. Sufficient therapists were recruited, but one was not suitable for this group of patients and dropped out of the study. Uniquely, her participants registered negative change scores and a homoeopathic adverse event. All therapists expressed frustration at the difficulty of making contact, the amount of last-minute cancellations, and the levels of non-attendance.

### Adverse events

The researchers concurred that one moderate Grade 2 event of worsening behaviour and two mild Grade 1 adverse events of itchy skin and increased aggression were reactions to nutritional supplements. One moderate Grade 2 event of a skin rash was considered a probable reaction to homoeopathic treatment.

## Discussion

The aim of the Sheffield Treatments for ADHD Research (STAR) project is to improve outcomes for children with ADHD by developing a facility for efficiently and objectively testing the long-term effectiveness of multiple interventions. This small-scale test suggests that the TwiCs design and procedures are feasible to achieve this aim with minor adjustments. The interventions trialled were sufficiently effective and acceptable according to feasibility parameters, and now require adequately powered testing. Although small effect sizes were found, these may have been influenced by therapist’s contacting strategies and attrition, and can be addressed.

Most feasibility criteria parameters were met: the primary outcome was sufficiently sensitive; recruitment procedures were satisfactory; outcome collection from carers worked well after the addition of an incentive, although could still be further improved, particularly concerning collection of outcomes from those who chose not to have treatment; the sample of participants recruited was broadly representative of those with ADHD; no serious adverse events attributable to treatment occurred; sufficient therapists were recruited although one was unsuitable for this population.

The methods used to obtain outcomes from teachers were not adequate and require improvement. Different and more approaches are needed to contact schools. Collection is important because blinded teacher outcomes provide objective assessments of behaviour in group settings, the majority of early year costs of ADHD are in education [17] and decision makers prioritise blinded results. There are issues with outcome collection from both carers and teachers. The concern with carer results is that they are unblinded and carers may be invested in treatment success which may affect their ratings [25]. The issue with teacher outcomes is that subtle changes may not be observed in busy classrooms, children may behave differently in structured environments, and collection of teacher outcomes are associated with procurement difficulties, completion by multiple teachers, and inability to collect outcomes during school holidays [2].

Both the interventions trialled are complex, comprising multiple, interacting components; they provide similar time, attention, and individually tailored advice; something to ingest between consultations; and are delivered by empathic practitioners. They differ in that implementation of dietary changes requires more effort than homoeopathic treatment; homoeopaths prefer attendance of both carer and child at consultations; and nutritional therapy is backed by explanations of mechanism and trials demonstrating the efficacy of some supplements and dietary approaches [24], whilst explanations of a mechanism for the action of homoeopathic medicines is not established, although four trials suggest the efficacy of individually tailored homoeopathic medicines [1, 9, 15, 19]. As befits a pragmatic trial, both interventions were trialled as experienced in clinical practice, and results reflect the effects of being offered this experience, not the efficacy of the ingested substances (homoeopathic medicines, nutritional supplements, or dietary inclusions/exclusions).

### Implications for future research

Some carers reported to their therapists that implementation of nutritional advice improved the nutrition of the whole family; and that implementation of homoeopathic treatment improved unexpected aspects such as gut dysbiosis, anxiety, eczema, and medication side effects. A future study might assess these anecdotal observations more systematically.

The TwiCs approach to conducting pragmatic trials can address important gaps in ADHD research, facilitate fast and efficient recruitment of participants, and provide useful information to stakeholders. Successful recruitment to the STAR cohort is likely to have been accomplished due to minimal commitment or delusion regarding interventions. However, the TwiCs approach uses a two-stage approach to informed consent, and participants, whilst easily recruited to the cohort (the first stage), were less easily retained at the second stage (the trial).

Greater refusal of the offer is to be expected using the TwiCs design compared with traditional RCT designs, where those not happy to accept a treatment or a placebo refuse participation prior to randomisation [33]. Whilst traditional RCTs often struggle to recruit sufficient participants (numbers declining are generally not recorded), they may better retain those they do recruit. However, information about acceptability is a useful feature of the TwiCs design, providing important information to stakeholders because: mainstream treatments for ADHD are not particularly acceptable and attrition and non-take up are a feature [16, 22]; information across trials with similar degrees of pragmatism can be useful; there is little point in having efficacious or effective interventions if they are not acceptable to their population; and uncertainty still remains regarding the effectiveness of the two mainstream intervention types (pharmaceutical medication and behavioural change programmes) in routine clinical practice.

The evidence base for child and adolescent mental health interventions to improve long-term mental health, is minimal [8, 14]. Reasons given are that relevant interventions are generally complex and multi-dimensional [13]; levels of comorbidity are high; and research expensive, labour intensive, and requiring specially trained staff [18]. The comparative effectiveness of mainstream interventions for ADHD is difficult to ascertain due to the use of different trial designs and samples [20, 28, 29], and their long-term effectiveness is also unknown. The TwiCs approach provides a potential solution to these issues. It is already being successfully implemented in five cohorts of children similar to the STAR ADHD cohort in being at risk of long-term negative outcomes (https://www.twics.global/use-of-the-design). Taking innovative pre-emptive approaches by developing cohorts and testing multiple interventions to try and improve outcomes is an emerging strength and potential of the design.

The STAR cohort now requires expanding, focusing on recruitment of those most at need: teenagers, hard to reach families, those with co-occurring autism, looked after children, those involved in criminality, and those with multiple co-morbidities. Improvement in collection of teacher outcomes and therapist’s contacting strategies are needed, by improving links with schools, increasing the number of reminders to teachers, and using multiple contacting modes to both carers and teachers.

The two piloted interventions now require testing in greater numbers. Future studies will need to address stakeholder concerns regarding the generalisability and variability of therapist-led interventions. However, both interventions appear safe, potentially effective, cheap to implement, and may be particularly useful: in the early years before pharmaceutical medication is permitted; for the 25%+ children with ADHD who cannot tolerate pharmaceutical medications; and for teenagers opting to discontinue their pharmaceutical medications. Further trials of other main and non-mainstream interventions are also planned.

## Conclusion

Children with ADHD are at risk of negative outcomes and in need of pre-emptive strategies implemented as early as possible. The STAR project demonstrated the feasibility and utility of the TwiCs approach to pragmatic RCT design for children with ADHD. It can make a useful contribution in the search to improve outcomes for those with ADHD.

## Abbreviations

ADHD: Attention deficit hyperactivity disorder
CGI: Conners Global ADHD Index
CHU 9D: Child Health Utility–9 Dimensions
CTCAE: Common Terminology Criteria for Adverse Events
Hom: Treatment by homoeopaths
HRQOL: Health-related quality of Life
ITT: Intention to treat
NT: Treatment by nutritional therapists
RCT: Randomised controlled trial
SPSS: Statistical Package for the Social Sciences
SMD: Standardised mean difference
STAR: Sheffield Treatments for ADHD Research
TwiCs: Trials within Cohorts
